# Partners in Practice: Primary Care Physicians Define the Role of Artificial Intelligence

**DOI:** 10.3390/healthcare13161972

**Published:** 2025-08-11

**Authors:** Dikla Agur Cohen, Anthony David Heymann, Inbar Levkovich

**Affiliations:** 1 Department of Family Medicine, The Rappaport Faculty of Medicine, Technion—Israel Institute of Technology, Haifa 3200003, Israel; 2 Clalit Health Services, Haifa and Western Galilee District, Haifa 3309101, Israel; 3 Department of Medical Education, Faculty of Medicine and Health Sciences, Tel Aviv University, Tel Aviv 6997801, Israel; 4 Meuhedet HMO, Tel Aviv 6812509, Israel; 5 Faculty of Education, Tel-Hai College, Upper Galilee 1220800, Israel

**Keywords:** artificial intelligence (AI), healthcare technology, human-centered AI, qualitative research, primary care, needs assessment

## Abstract

Background: Artificial intelligence (AI) shows strong potential to transform primary care by streamlining workflows, improving diagnostics, and enhancing patient outcomes. However, integration faces barriers, including PCPs’ concerns about workflow disruptions, reliability, and loss of human connection. This study explored PCPs’ perspectives and challenges around AI integration in primary care to inform the development of practical, human-centered tools. Method: This qualitative study included four focus groups (n = 40), comprising PCPs, residents, and AI developers, in December 2024. Sessions were recorded, transcribed, and analyzed using thematic analysis. Three main themes emerged: (1) *From Frustration to Innovation*: PCPs’ experiences with current technological gaps and their vision for improved support; (2) *The Integration Paradox*: tensions in embedding AI while safeguarding care quality; and (3) *Beyond Basic Automation*: future solutions that preserve clinical judgment. Result: Key findings emphasized the need for incremental AI adoption, starting with administrative tasks and progressing to clinical decision support, with systems acting as “silent partners” to enhance rather than replace human judgment. PCPs see AI as a promising way to reduce administrative burden and improve care quality but stress the need for human-centered design that protects the doctor–patient relationship. Conclusion: Successful integration requires addressing workflow compatibility, ethical concerns, and preserving clinical autonomy through collaborative development.

## 1. Introduction

The rapid advancement of artificial intelligence (AI) has great potential to transform healthcare, especially primary care. Integrating AI can enhance efficiency, improve patient outcomes, and alleviate providers’ burden [1]. By utilizing extensive data, AI can revolutionize diagnostics, treatment planning, and patient monitoring to deliver personalized care and optimize clinical decision making [2,3,4]. A major challenge for primary care physicians (PCPs) is the administrative burden, which consumes 15–25% of their work time and is dedicated to nonclinical tasks [5,6], hinders patient care, and contributes to burnout [7,8,9]. AI technologies, which automate clinical visits and utilize electronic health record data to assist with diagnostics and clinical note drafting, help to alleviate these pressures by freeing up time for patient interaction and reducing cognitive overload [10,11].

Despite AI’s potential, its integration into primary care is challenging, as technologies must align with clinical realities for effective adoption [3,12]. While PCPs generally view AI positively, concerns regarding its accuracy, safety, biases, and impact on workflows, equity, and the doctor-patient relationship persist. This highlights the need for AI tools to serve as supportive assistants that can integrate seamlessly into existing workflows [13,14].

A systematic review emphasized the need for greater physician trust in AI, noting that its perceived value relies on seamless integration and usability [15]. Primary care physicians acknowledge AI’s potential for healthcare task management but stress that it should augment rather than replace their roles [16]. A July 2024 survey (conducted to reflect the most up-to-date AI developments and clinical realities in primary care) found that many AI tools are poorly adapted to primary care workflows, limiting their effectiveness and adoption [17]. Researchers assert that in order to be meaningful in clinical practice, AI solutions must reduce administrative burdens, particularly EHR-related ones, and address physician burnout [6,18,19,20]. Research involving deliberative dialogue with PCPs has identified several barriers to AI implementation, including system and data readiness issues, such as interoperability, data security, data quality, the potential for bias and inequity, and the need for regulation [21,22]. While most PCPs believe that AI will enhance healthcare delivery efficiency, its development must involve collaboration with providers to meet their specific needs [23,24]. Previous studies from the UK, USA, and Australia have reported similar tensions in AI implementation, including physician skepticism, integration into workflows, and ethical challenges [23,24]. However, few have explored these issues through a participatory, user-centered lens specific to primary care physicians. The current study builds on this international work while offering an in-depth exploration of the Israeli context.

There is still, however, a lack of in-depth, user-centered research exploring the specific needs, preferences, and practical challenges that PCPs face when integrating AI into daily practice. Existing research often lacks a structured framework for understanding how AI tools can be tailored to physicians’ real-world demands and workflows to provide benefits that are more than just theoretical. This study aimed to fill this gap by directly engaging PCPs in a qualitative exploration of AI integration through deep discussion in four diverse focus groups. This participatory approach provides practical, firsthand insights for effective AI adoption and offers a roadmap for developing AI solutions that align with PCPs’ needs rather than add to their workload.

This study addresses a notable gap in the literature by focusing specifically on the lived experiences of primary care physicians (PCPs) navigating AI implementation. Unlike many previous studies that rely on generalized clinician perspectives, our research centers on the nuanced, practice-based needs of PCPs, whose workflow complexities, ethical concerns, and patient relationships present unique challenges for AI integration.

## 2. Materials and Methods

### 2.1. Study Design

This study employed a qualitative phenomenological approach [25] to explore PCPs’ experiences and perceptions regarding integrating AI into primary care. The phenomenological approach focuses on uncovering the meaning of the participants’ experiences and the essence of the phenomenon under investigation.

Focus groups provided in-depth insights into PCPs’ interactions with and expectations of AI in clinical practice. An anonymous online survey was conducted beforehand to help design the focus group discussion framework and ensure that the topics reflected real-world concerns.

### 2.2. Participants

This study involved 40 participants divided into four focus groups of 8–12 participants each. Recruitment was conducted through targeted outreach on professional social media platforms and digital health forums. Inclusion criteria comprised board-certified PCPs, family medicine or pediatric residents, and healthcare AI developers. Exclusion criteria included inability to attend a full session or refusal to provide informed consent. A purposive sampling strategy was employed to ensure diversity in professional roles, levels of seniority, and familiarity with AI technologies. Those unwilling to provide informed consent or unable to attend the entire session were excluded to ensure a relevant and consistent dataset. The four focus groups differed concerning the diverse expertise of their participants: The first group consisted of board-certified PCPs, the second of PCPs involved in AI implementation in the Israeli Health Maintenance Organization, the third of family medicine and pediatrics residents, and the fourth of AI developers and healthcare technology professionals.

### 2.3. Research Tools

Focus group discussions were conducted using a structured guide designed to provide a clear framework while allowing flexibility to encourage natural dialogue and meaningful self-expression among participants. The discussion guide (Table 1) was developed based on qualitative data from an anonymous online survey, which helped refine key themes but was not used as a standalone data source.

### 2.4. Survey

An online survey was distributed in July 2024 to PCPs via professional social media platforms. The objective was to collect information to help design the focus group discussion topics. The survey included three open-ended questions: (1) If no technological or financial constraints existed, what technology would you ideally want for yourself and your patients? (2) What improvements to existing systems would you most like to implement in your work? (3) What are your concerns about integrating AI technology into your future work?

Survey responders (N = 60) were 61% male and 39% female, and the majority of them were specialists (71%), with 73% having over ten years of experience. The insights from their responses were instrumental in constructing the interview guide, which served as a foundation for the focus group discussions. The survey was not a standalone data collection tool but served as a preparatory step to inform the qualitative inquiry in a user-centered manner. Rather than functioning as an independent data source, it guided development of the focus group discussion framework, ensuring alignment with current clinical realities and the concerns voiced by PCPs.

### 2.5. Focus Groups

The focus groups were conducted by the researchers, A.D.H and D.A.C, one male and one female, both holding M.D. degrees with extensive qualitative research experience. The sessions took place via Zoom between September and December 2024. Sessions lasted approximately 75 min each and were audio- and video-recorded with the participants’ consent. The discussion guide included open-ended questions addressing AI’s potential benefits and challenges and the integration processes of AI in primary care workflows. The facilitators ensured an inclusive environment that promoted diverse perspectives. The data analysis process was completed within two months, ensuring a thorough and timely interpretation of the findings.

### 2.6. Data Collection

All focus group recordings were transcribed verbatim to safeguard participants’ confidentiality. Field notes taken during the sessions supplemented the transcripts, enhancing the richness and depth of the dataset. Discussions were structured in order to delve into key themes while allowing for the emergence of novel insights.

### 2.7. Data Analysis

Thematic analysis within a phenomenological framework [26] explored recurring themes and patterns related to integrating AI into primary care. The focus group discussions were transcribed by a professional English-speaking translator, and the authors carefully reviewed and edited the resulting transcripts to ensure accuracy. As the focus groups were conducted in Hebrew, translation to English was required to facilitate joint analysis and manuscript preparation within a multilingual research team. Bilingual researchers cross-checked the translated transcripts with the original recordings to ensure fidelity to the participants’ meanings. A qualitative analysis was conducted manually with line-by-line coding applied to the data using the “TAGUETTE” software (version 1.4.1) [27]. Codes were developed deductively, guided by the study’s research questions, and inductively, allowing themes to emerge organically from the data. This dual approach ensured a comprehensive and grounded analysis.

The coding process involved three independent researchers who reviewed the data. Each researcher used a combination of deductive and inductive coding to identify initial codes and patterns. These codes were then iteratively reviewed and organized into broader thematic categories. Ongoing discussions between the researchers helped ensure consistency, resolve discrepancies, and refine the coding framework. The final themes were collaboratively identified and aligned with the study’s objectives to provide a thorough understanding of the phenomenon under investigation, interpreted in line with the study objectives and supported by illustrative quotes from the data. Intercoder reliability was established through iterative consensus meetings among the three coders, ensuring consistency in the application of codes and thematic categorization. Reliability was enhanced through intercoder agreement and triangulation with additional data sources [28]. This iterative process also incorporated reflexivity, creating a dynamic cycle of data collection, analysis, and planning. Insights from the initial analysis informed modifications to the subsequent focus group questions, enabling researchers to refine and deepen their inquiries. Similar codes were consolidated into cohesive themes using an analytical framework. The final themes were determined through a collaborative review and discussion by the research team. Interpretations were carefully explored to ensure they provided meaningful insights that aligned with the study’s goals.

### 2.8. Ethical Considerations

This study was conducted in accordance with the ethical principles outlined in the Declaration of Helsinki and was approved by the Institutional Review Board (IRB) of the Rappaport Faculty of Medicine, Technion-Israel Institute of Technology (IRB #2024-060). Participation in the study was entirely voluntary, with no financial or material incentives offered to participants. Prior to participation, all individuals received a detailed explanation of the study’s aims and procedures and signed an informed consent form. Participants were assured of their right to withdraw from the study at any point without any consequences. Confidentiality and anonymity were strictly maintained throughout all stages of the research. All data were securely stored in encrypted digital files and were accessible only to members of the research team directly involved in the study.

## 3. Results

The focus groups included 40 participants, with males being slightly predominant (n = 22; 55.0%) compared with females (n = 18; 45.0%). In terms of the participants’ main job, the majority were family practitioners (n = 30; 75.0%), followed by pediatricians (n = 7; 17.5%) and developers (n = 3; 7.5%), and as for career stage, most participants held a board certificate (n = 25; 62.5%), while the others were residents (n = 12; 30.0%) or seniors who were not working as physicians (n = 3; 7.5%). Finally, the majority of participants reported moderate knowledge of AI (n = 23; 57.5%), with fewer participants indicating no experience (n = 8; 20.0%) or expert knowledge (n = 9; 22.5%) (Table 2).

**Figure 1:**
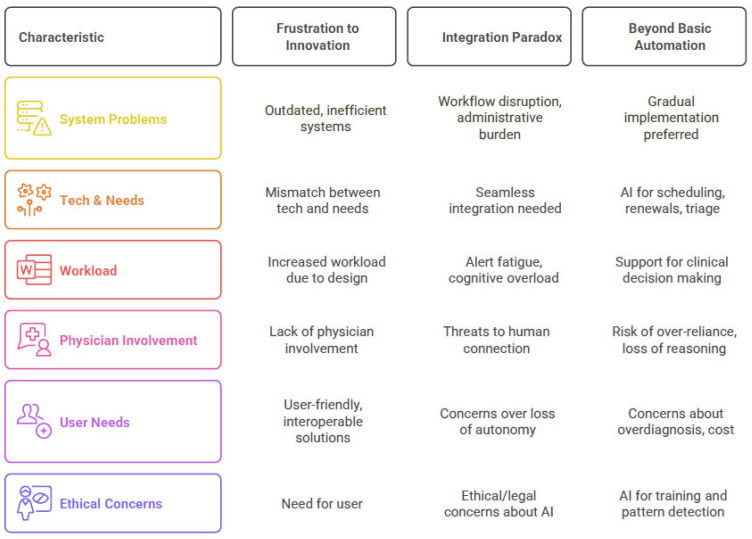
Main themes and key categories.

Three main themes emerged from the focus group regarding integrating technological systems into primary care (Figure 1).

The first theme, From Frustration to Innovation, captures physicians’ experiences with current technological limitations and their vision for more advanced support systems. The second theme, The Integration Paradox: When Innovation Meets Clinical Reality—Challenges and Opportunities in Workflow Integration, explores the complex challenges of incorporating new technologies into existing clinical workflows while maintaining quality patient care. The final theme, Beyond Basic Automation, delves into specific solutions and future directions physicians propose while acknowledging their concerns about maintaining clinical judgment and the human aspect of medical care.

### 3.1. Theme 1: From Frustration to Innovation: The Need for Advanced Technological Support Systems

Healthcare providers have consistently emphasized significant limitations in current computerized support systems, highlighting a critical gap between existing capabilities and their professional needs. Participants expressed frustration with outdated systems that failed to match the pace of technological advancement in other sectors:

“I’m not worried. Our healthcare systems are outdated compared with today’s technology”.

[Focus Group (FG) 1]

This frustration extends beyond technological limitations to increased workloads and system inefficiency concerns. Many physicians reported that the current systems often create additional burdens rather than alleviate them:

“I’m concerned about technologies that increase my workload instead of reducing it, as they often require me to work for the systems”.

(FG3)

The impact of inadequate systems appears particularly pronounced in daily clinical operations, where physicians develop workarounds to compensate for the system limitations. This highlights the urgent need for more sophisticated and user-friendly solutions:

“To address these limitations, systems should incorporate advanced UI/UX design and robust interoperability, enhancing user experience and ensuring seamless integration in primary care”.

(FG2)

Furthermore, physicians expressed frustration over their lack of involvement in ideating, developing, and implementing new technologies, feeling sidelined as end users. Indeed, they are frequently required to adopt tools designed without their input, which may not align with the reality of clinical practice:

“We are handed new systems without being asked what we actually need, and then we’re the ones who have to make them work”.

(FG1)

Notably, early-career participants, including residents, were especially vocal about their limited role in the development of clinical technologies. They described a disconnect between the tools provided and the realities of frontline practice, emphasizing the need for systems that reflect their evolving workflows and responsibilities.

### 3.2. Theme 2: The Integration Paradox: When Innovation Meets Clinical Reality—Challenges and Opportunities in Workflow Integration

The critical importance of seamless integration between automated support systems and existing clinical workflows emerged as a central theme. Physicians emphasized that technological solutions must enhance, rather than complicate, their ability to provide patient care:

“We need an interface that seamlessly communicates in real-time with hospital systems and imaging centers, ensuring smooth coordination rather than adding more steps to our workflow”.

(FG2)

Instead of easing their workload, these technologies often increase administrative burdens, disrupt workflows, and add complexity, further exacerbating their sense of disempowerment and dissatisfaction:

“No one asks for our input before implementation. We find ourselves struggling with technologies that not only fail to ease our workload but also add unnecessary burdens and complexity”.

(FG1)

They noted that AI systems should offer assistance and act as silent partners, stepping in when needed without drawing too much attention from the patient. Another significant concern is alert fatigue, which occurs when excessive notifications and prompts, as is seen in some current systems, lead to distraction and reduced efficiency. A significant concern among the participants was maintaining the human aspect of healthcare while incorporating technological advances. Their responses stressed the importance of balancing automation with personal care:

“Medical practice may become overly technical, compromising the human connection crucial for quality healthcare. Standardized protocols may overlook individual needs, as people often don’t fit into predefined categories”.

(FG4)

Maintaining clinical autonomy while integrating AI systems also emerged as a crucial concern. Physicians expressed concern about the potential loss of professional judgment and decision making authority:

“AI should support, not override, clinical judgment—integrating real-time patient data with guidelines can aid decision making, but the final call must remain with the physician”.

(PG4)

“[I’m concerned about] losing control, as AI-driven recommendations or managerial directives may not align with good medicine principles”.

(PG2)

Nevertheless, participants anticipated that AI-driven systems will assist clinical decision making by providing recommendations based on current guidelines. The referral process will be automated, with referrals and forms generated in real time, requiring only the physician’s confirmation. Some variation in concerns was observed between more and less experienced clinicians. Senior physicians often emphasized threats to professional autonomy and ethical responsibility, while junior participants tended to focus on practical workflow disruptions and uncertainty about the scope of AI’s role in decision making. These differing concerns reflect how career stage may influence attitudes toward AI integration.

Participants expressed significant concerns about the legal responsibilities and ethical implications of using AI in healthcare. The tension between trusting clinical experience, pressure to adhere to AI recommendations, and potential medico-legal risks may discourage physicians from deviating from AI-driven advice. In such cases, physicians may face scrutiny or be required to justify their decisions, raising fears of liability and loss of autonomy:

“Suppose the AI suggests an advanced imaging test that I think is unnecessary given the patient’s clinical presentation. In such a case, I may need to justify not following its suggestion, which can feel like defending my expertise against a machine”.

(FG1)

### 3.3. Theme 3: Beyond Basic Automation: Solutions and Future Directions

The participants envisioned sophisticated automated systems that could enhance their clinical practice while preserving their professional judgment. Their suggestions focused on practical solutions to the common challenges in primary care:

“An AI system that can manage all prescription renewals and recommend necessary lab tests for ongoing treatment. It can also analyze patient medication and supplement interactions, providing accurate and up-to-date recommendations”.

(FG4)

Developers emphasized an incremental approach to AI implementation, suggesting a progression from basic administrative tasks to clinical decision making functions. This gradual integration would prioritize automated processes like prescription renewals and triage while enabling patient self-management of routine tasks through dedicated applications. The proposed solutions emphasize the importance of evidence-based personalized care integrating multiple data sources and clinical guidelines:

“[The integration should start from tasks] that do not involve physicians, like automation of administrative processes. For example, a smart scheduling system could manage appointments and, over time, learn what works best without requiring physician input”.

(FG1)

The participants advocated for intelligent scheduling systems that adapt to real-time clinical needs, automatically adjusting appointment durations based on case complexity and patient history, thus optimizing resource allocation and clinical efficiency. However, concerns about the potential negative impacts of over-reliance on technology were also prominent, particularly regarding clinical reasoning skills and professional development:

“I fear relying on technology too much, losing my clinical thinking, and falling into automatic actions—risking the loss of clinical reasoning, much like when using driving navigation apps”.

(FG3)

Differences also emerged based on participants’ prior experience with AI. Those with more hands-on exposure to AI systems tended to offer detailed, concrete suggestions for integration and training. In contrast, less experienced participants were more cautious and emphasized the importance of transparency and simplicity in early-stage implementation. These differences suggest that familiarity with AI may shape clinicians’ readiness to adopt more advanced functionalities. Beyond administrative functions, participants envisioned AI systems capable of analyzing subtle communication patterns during virtual consultations to identify potential underlying conditions, particularly in mental health and cognitive domains. They also highlighted AI’s potential role in continuing medical education through simulated cases and real-time feedback mechanisms. Furthermore, physicians expressed concern that AI recommendations could lead to overdiagnosis by suggesting unnecessary tests or treatments. This could expose patients to avoidable risks, increase healthcare costs, and burden the system with interventions that may not improve outcomes:

“The AI might flag every minor abnormality as something to investigate further. We could end up ordering tests and treatments for things that would never have caused harm, unnecessarily turning healthy people into patients”.

(FG2)

## 4. Discussion

This study investigated the needs of PCPs concerning AI technology, offering crucial insights into the future of healthcare. The findings underscore several key areas in which AI could significantly enhance clinical practice while highlighting essential concerns that must be addressed. Focus group discussions provided in-depth, real-world perspectives that revealed both opportunities and challenges in the successful integration of AI within primary care settings.

In the current study, physicians consistently expressed a strong need for AI-assisted tools to reduce administrative burdens, particularly those related to documentation, transcription, and routine tasks such as prescription renewals and referrals. This demand aligns with recent literature that indicates that administrative tasks consume a significant portion of physicians’ time, contributing to burnout and reduced patient care quality [5,11,13]. Administrative tasks should, therefore, be the primary focus of AI integration in primary care, and focus group discussions highlighted the importance of designing AI systems that integrate seamlessly into clinical workflows without adding extra layers of complexity [29,30]. Participants emphasized that AI should act as a silent partner, aiding clinical practice without overwhelming its users. This perspective is supported by research that shows that when it comes to medical decision making tasks, AI–human collaborations often outperform AI or humans working alone [16,29,30]. Participants also raised concerns about alert fatigue, where excessive notifications disrupt workflow and reduce efficiency. To avoid this, AI tools must be designed to streamline tasks while minimizing distractions [31,32]. Furthermore, participants stressed the importance of a user-centered approach, advocating for gradual implementation guided by practitioners, with continuous feedback and iterative improvements based on physician input [11,33]. Developers emphasized the potential of AI in incremental implementation and recommended focusing on administrative and operational tasks as entry points. They proposed starting with triage systems, automating lab result analysis, and prescription renewals tools that reduce workload without interfering with clinical decision making [34].

The findings of the present study demonstrate several innovative uses of AI that serve to extend patient care. Participants discussed the potential of AI-driven chatbots to manage repetitive patient queries and the development of smart scheduling systems that will predict and adapt appointment duration based on patient history and case complexity. These innovations suggest that AI can support care beyond in-person visits, helping streamline administrative processes and free time for more patient-centered interactions [12,16]. Developers added that data consolidation is crucial for these innovations, in which AI will, among other things, enable the integration of fragmented medical records to improve care coordination and continuity [2,6]. Indeed, they highlighted the role of AI in ensuring data quality and creating a foundation for advanced applications. Integrating these points into the discussion highlights new avenues through which AI can enhance primary care efficiency and quality by addressing physician workload and patient needs [4,33].

While participants did not refer to specific AI tools by name, several of their comments reflect expectations and concerns associated with current large language models. These include systems such as GPT-based tools that assist with documentation, summarization, and recommendation generation [16,34]. These models are increasingly used in administrative and diagnostic support but still present challenges in explainability, bias, and accountability [31,33]. In contrast, agentic AI systems focus on autonomous task execution and adaptive decision making, raising further concerns about control, legal responsibility, and the erosion of clinical judgment [6]. These distinctions are important for understanding the technologies participants may have implicitly referenced and emphasize the need for alignment between system capabilities and physicians’ expectations of transparency, empathy, and safety [35].

A critical area of concern raised by participants was the potential for overdiagnosis due to AI systems possibly recommending unnecessary tests, treatments, and follow-ups. Such outcomes can lead to significant economic, medical, and legal repercussions [29,35]. Participants cautioned that over-reliance on AI can escalate healthcare costs, expose patients to unnecessary risks, and increase the likelihood of malpractice claims [31,36]. These insights underscore the need for stringent regulation and validation of AI systems to ensure that they prioritize patient safety and are sensitive to the nuances of clinical decision making, thereby avoiding overuse and inappropriate intervention [30,37]. Developers emphasized the importance of liability frameworks, cautioning that tools must clearly delineate physician supervision to mitigate risks. They also pointed out the need for algorithms to incorporate feedback loops to refine recommendations over time [35,36].

The focus group discussions held as part of this study strongly reaffirmed the essential role of human interaction in healthcare, emphasizing that AI, while capable of mimicking empathy to some extent, cannot replace the therapeutic relationship between patients and physicians [34]. This aligns with existing literature that highlights the importance of maintaining empathy and human connection, even as AI becomes more prevalent in healthcare settings [12,37]. Participants from the developers’ group proposed AI tools that act as a communication facilitator, ensuring that information flows efficiently between patients and providers [33]. Participants, particularly younger physicians, expressed concerns that a growing reliance on AI could erode the personal aspects of care, potentially leading to a loss of job satisfaction and re-evaluation of their commitment to the profession [16,36]. These concerns have significant implications, suggesting that AI should be designed to support and enhance rather than replace interpersonal interactions, which are central to healing [30].

The anxiety surrounding job security, particularly among younger participants, reflects broader concerns regarding the ethical implications of AI in clinical practice. Participants feared that AI would reduce the autonomy of family practitioners or even replace them, posing risks to their professional roles. This sentiment calls for a balanced approach to AI deployment that enhances the skills and autonomy of healthcare professionals, ensuring that technological advancements support their roles rather than undermine them [19,36]. Addressing these concerns adds depth to the discussion on the responsible implementation of AI, emphasizing the need for ethical guidelines that preserve the physician’s role as a central figure in patient care [30,35]. In this context, trust in AI systems becomes essential not only in terms of their accuracy but also in ensuring that their integration reinforces, rather than weakens, clinicians’ sense of purpose and job satisfaction [16].

The participants also highlighted AI’s potential to enhance the education and continuous learning of healthcare professionals. They envisioned AI systems that can simulate patient interactions, generate case scenarios, and provide real-time feedback, facilitating ongoing skill enhancement. Developers proposed incorporating AI into educational platforms to train physicians on the use of new technologies, including AI-driven simulations replicating real-world complexities that enable facilitators to enhance decision making skills in low-risk environments [30,31]. Integrating these insights into the discussion broadens the perspective on AI’s role in education and training, suggesting that it can be a powerful tool not only for improving clinical efficiency but also for supporting lifelong learning and professional development among healthcare providers [35,36]. Additionally, integrating AI into training environments offers opportunities to foster ethical awareness and strengthen physicians’ confidence in navigating complex clinical–technological interfaces [37]. These findings echo conclusions from international studies conducted in the UK, USA, and Australia, which similarly underscore the importance of clinician trust, workflow integration, and ethical safeguards in the implementation of AI tools in primary care [15,22,23]. The current study contributes to this literature by offering a user-centered, context-specific perspective rooted in the Israeli healthcare system, thereby enriching the global understanding of the challenges and facilitators in AI adoption.

### Limitations

First, the study sample consisted primarily of Israeli PCPs, which may limit the generalizability of the results to other countries or healthcare systems. AI adoption, technological infrastructure, and regulatory frameworks vary significantly among regions, potentially influencing physicians’ attitudes and needs. Including international participants would strengthen the study’s global applicability. A more diverse global sample would have provided broader insights. Second, the data were collected using focus groups, which may be affected by biases such as social desirability and recall bias. Participants may have expressed views they believed were expected or more socially acceptable, potentially limiting the depth of insight into their genuine concerns or experiences. Moreover, the voluntary nature of participation and recruitment through professional networks may have introduced a self-selection bias, potentially over-representing participants with greater interest or familiarity with AI technologies. Third, the use of open-ended questions allowed for rich qualitative data but also made it challenging to quantify specific responses. The subjective interpretation of responses during the coding process may have introduced unintended biases, although efforts were made to minimize this bias through independent coding and triangulation. In addition, although the sample included participants from various professional roles, the group was relatively homogeneous in terms of geographic and healthcare system context, which may limit the broader applicability of the findings. Furthermore, the study did not perform subgroup analyses (e.g., comparing residents with board-certified physicians or participants with varying levels of AI exposure). This limits the ability to detect potentially divergent viewpoints across roles, experience levels, or familiarity with AI, and may have masked important nuances in how AI integration is perceived. Additionally, although the sample included participants from diverse roles and levels of experience, the study did not perform subgroup analyses (e.g., by role, seniority, or prior AI exposure), which may have masked differences in perceptions across groups. Additionally, the study relied solely on focus group data. Incorporating additional sources, such as individual interviews or direct clinical observations, could have provided deeper insight into physicians’ behaviors and decision making. While beyond the scope of this study, such approaches are recommended for future research. Finally, AI technology has evolved and is continuing to evolve rapidly, and when AI tools are fully developed and implemented, new advancements or challenges may emerge, potentially altering physicians’ perspectives and needs. The dynamic nature of AI requires continuous re-evaluation of healthcare providers’ needs.

## 5. Conclusions

The findings of this study illustrate the potential benefits and challenges of integrating AI with primary care. By identifying the specific needs, preferences, and barriers PCPs face, this study highlights pathways to develop AI tools that reflect real-world clinical demands. AI technologies can enhance practice by reducing administrative burden, improving diagnostic support, and extending patient care. However, thoughtful implementation is critical to ensure that these tools align with PCPs’ workflows and address challenges, such as overdiagnosis, loss of human connection, and ethical concerns about job security and autonomy. Incremental implementation, beginning with tasks like triage, data consolidation, and administrative support, can demonstrate immediate value while fostering trust and usability.

To enhance practical adoption, we suggest initiating implementation in well-defined areas such as prescription renewals, referral generation, and lab result interpretation. These low-risk use cases allow physicians to gain confidence in AI while maintaining clinical oversight. Institutional leaders and policymakers can facilitate adoption by providing structured training programs, establishing clear validation protocols, and ensuring transparent governance frameworks that protect professional autonomy and support physician engagement. This study underscores the importance of designing AI systems as technological innovations and tools deeply informed by the realities of primary care, ensuring their meaningful integration into healthcare practice.

## Figures and Tables

**Table 1:** Focus group interview guide.

If there were no technological or financial constraints, what technology would you ideally want for yourself and/or your patients?
How might AI change your daily tasks and overall workflow?
What improvements to existing healthcare systems would you most like to see facilitated by AI?
What are your concerns about integrating AI technologies into your future work?
What limitations or obstacles do you foresee in implementing AI tools in primary care settings?
How might AI influence ethical decision making in patient care?
What safeguards are essential when incorporating AI into clinical workflows?
What specific support or training do physicians need in order to integrate AI into their practices effectively?

**Table 2:** Data of focus group participants (N = 40).

Focus Group	No. 1 (N = 12)	No. 2 (N = 10)	No. 3 (N = 10)	No. 4 (N = 8)
	8 September	14 October	29 October	24 December
Main Job				
FP	8 (66.66%)	10 (100%)	8 (80%)	4 (50%)
Pediatrician	4 (33.34%)	-	2 (20%)	1 (12.5%)
Developer	-	-	-	3 (37.5%)
Career Stage				
Resident	2 (16.67%)	-	10 (100%)	-
Board certificate	10 (83.33%)	10 (100%)	-	5 (62.5%)
Senior expert/	-	-	-	3 (37.5%)
Professional				
Gender				
Female	4 (33.34%)	4 (40%)	5 (50%)	5 (62.5%)
Male	8 (66.66%)	6 (60%)	5 (50%)	3 (37.5%)
Age	40–60 yr	45–65 yr	30–40 yr	40–55 yr
AI Experience				
No experience	5 (41.66%)	-	3 (30%)	-
Moderate	7 (58.34%)	9 (90%)	7 (70%)	-
Expert	-	1 (10%)	-	8 (100%)

## Data Availability

The data presented in this study are available on request from the corresponding author.

## Abbreviations

The following abbreviations are used in this manuscript:

AI	Artificial intelligence
PCPs	Primary care physicians

## References

[1] Shaheen M.Y. (2021). Applications of Artificial Intelligence (AI) in Healthcare: A Review. Sci. Prepr..

[2] Bajwa J., Munir U., Nori A., Williams B. (2021). Artificial Intelligence in Healthcare: Transforming the Practice of Medicine. Future Healthc. J..

[3] Davenport T., Kalakota R. (2019). The Potential for Artificial Intelligence in Healthcare. Future Healthc. J..

[4] Habehh H., Gohel S. (2021). Machine Learning in Healthcare. Curr. Genom..

[5] Zacay G., Adler L., Schonmann Y., Azuri J., Yehoshua I., Vinker S., Heymann A.D., Afek S., Cohen A.G., Green I. (2024). “A Day in the Life”—Telemedicine in Family Medicine and Its Relationship with Practicing Physicians’ Satisfaction: A Cross-Sectional Study. Isr. J. Health Policy Res..

[6] Rodríguez J.E., Lussier Y. (2025). The AI Moonshot: What We Need and What We Do Not. Ann. Fam. Med..

[7] Demers E. How Do Physicians Find Time to Provide Quality Care?. https://www.medicaleconomics.com/view/how-do-physicians-find-time-to-provide-quality-care-.

[8] Pastoor C. Can Artificial Intelligence Transform Primary Care?. https://www.elationhealth.com/resources/blogs/can-artificial-intelligence-transform-primary-care.

[9] Himmelstein D.U., Woolhandler S., Almberg M., Fauke C. (2017). The U.S. Health Care Crisis Continues: A Data Snapshot. Int. J. Health Serv..

[10] Seneviratne M.G., Shah N.H., Chu L. (2020). Bridging the Implementation Gap of Machine Learning in Healthcare. BMJ Innov..

[11] Van Buchem M.M., Boosman H., Bauer M.P., Kant I.M.J., Cammel S.A., Steyerberg E.W. (2021). The Digital Scribe in Clinical Practice: A Scoping Review and Research Agenda. npj Digit. Med..

[12] Lin S.Y., Mahoney M.R., Sinsky C.A. (2019). Ten Ways Artificial Intelligence Will Transform Primary Care. J. Gen. Intern. Med..

[13] Allen M.R., Webb S., Mandvi A., Frieden M., Tai-Seale M., Kallenberg G. (2024). Navigating the Doctor-Patient-AI Relationship—A Mixed-Methods Study of Physician Attitudes toward Artificial Intelligence in Primary Care. BMC Prim. Care.

[14] Olaye I.M., Seixas A. (2023). The Gap between AI and Bedside: Participatory Workshop on the Barriers to the Integration, Translation, and Adoption of Digital Health Care and AI Startup Technology into Clinical Practice. J. Med. Internet Res..

[15] Ayorinde A., Mensah D.O., Walsh J., Ghosh I., Ibrahim S.A., Hogg J., Peek N., Griffiths F. (2024). Healthcare Professionals’ Experience of Using Artificial Intelligence: A Systematic Review with Narrative Synthesis (Preprint). J. Med. Internet Res..

[16] Waheed M.A., Liu L. (2024). Perceptions of Family Physicians about Applying AI in Primary Health Care: Case Study from a Premier Health Care Organization. JMIR AI.

[17] Impact A. 70% of Clinicians Agree on AI’s Promise to Increase Efficiencies in Care Delivery, Finds Elation Health’s Latest Survey Results. https://www.elationhealth.com/resources/elation-health-ehr/ai-survey-2.

[18] Menchaca J.T. (2025). For AI in Primary Care, Start with the Problem. Ann. Fam. Med..

[19] Mainous A.G. (2022). Will Technology and Artificial Intelligence Make the Primary Care Doctor Obsolete? Remember the Luddites. Front. Med..

[20] Arndt B.G., Beasley J.W., Watkinson M.D., Temte J.L., Tuan W.-J., Sinsky C.A., Gilchrist V.J. (2017). Tethered to the EHR: Primary Care Physician Workload Assessment Using EHR Event Log Data and Time-Motion Observations. Ann. Fam. Med..

[21] Darcel K., Upshaw T., Craig-Neil A., Macklin J., Gray C.S., Chan T.C.Y., Gibson J., Pinto A.D. (2023). Implementing Artificial Intelligence in Canadian Primary Care: Barriers and Strategies Identified through a National Deliberative Dialogue. PLoS ONE.

[22] Razai M.S., Al-bedaery R., Bowen L., Yahia R., Chandrasekaran L., Oakeshott P. (2024). Implementation Challenges of Artificial Intelligence (AI) in Primary Care: Perspectives of General Practitioners in London UK. PLoS ONE.

[23] Yang Z., Silcox C., Sendak M., Rose S., Rehkopf D., Phillips R., Peterson L., Marino M., Maier J., Lin S. (2022). Advancing Primary Care with Artificial Intelligence and Machine Learning. Healthcare.

[24] Liyanage H., Liaw S.-T., Jonnagaddala J., Schreiber R., Kuziemsky C., Terry A.L., de Lusignan S. (2019). Artificial Intelligence in Primary Health Care: Perceptions, Issues, and Challenges. Yearb. Med. Inform..

[25] Vagle M.D. (2018). Crafting Phenomenological Research.

[26] Guest G., MacQueen K.M., Namey E.E. (2012). Applied Thematic Analysis.

[27] Rampin R., Rampin V. (2021). Taguette: Open-Source Qualitative Data Analysis. J. Open Source Softw..

[28] Creswell J., Poth C. (2018). Qualitative Inquiry and Research Design: Choosing Among Five Approaches.

[29] Badal K., Lee C.M., Esserman L.J. (2023). Guiding Principles for the Responsible Development of Artificial Intelligence Tools for Healthcare. Commun. Med..

[30] Alowais S.A., Alghamdi S.S., Alsuhebany N., Alqahtani T., Alshaya A., Almohareb S.N., Aldairem A., Alrashed M., Saleh K.B., Badreldin H.A. (2023). Revolutionizing Healthcare: The Role of Artificial Intelligence in Clinical Practice. BMC Med. Educ..

[31] Karalis V.D. (2024). The Integration of Artificial Intelligence into Clinical Practice. Appl. Biosci..

[32] Wan P.K., Satybaldy A., Huang L., Holtskog H., Nowostawski M. (2020). Reducing Alert Fatigue by Sharing Low-Level Alerts with Patients and Enhancing Collaborative Decision Making Using Blockchain Technology: Scoping Review and Proposed Framework (MedAlert). J. Med. Internet Res..

[33] Seneviratne M.G., Li R.C., Schreier M., Lopez-Martinez D., Patel B.S., Yakubovich A., Kemp J.B., Loreaux E., Gamble P., El-Khoury K. (2022). User-Centred Design for Machine Learning in Health Care: A Case Study from Care Management. BMJ Health Care Inform..

[34] Levkovich I. (2025). Is Artificial Intelligence the next Co-Pilot for Primary Care in Diagnosing and Recommending Treatments for Depression?. Med. Sci..

[35] Kerasidou A. (2020). Artificial Intelligence and the Ongoing Need for Empathy, Compassion and Trust in Healthcare. Bull. World Health Organ..

[36] Rogers W.A., Draper H., Carter S.M. (2021). Evaluation of Artificial Intelligence Clinical Applications: Detailed Case Analyses Show Value of Healthcare Ethics Approach in Identifying Patient Care Issues. Bioethics.

[37] Crossnohere N.L., Elsaid M., Paskett J., Bose-Brill S., Bridges J.F.P. (2022). Guidelines for Artificial Intelligence in Medicine: Literature Review and Content Analysis of Frameworks. J. Med. Internet Res..

